# The effect of endovascular baroreflex amplification on central sympathetic nerve circuits and cerebral blood flow in patients with resistant hypertension: A functional MRI study

**DOI:** 10.3389/fnimg.2022.924724

**Published:** 2022-07-25

**Authors:** Eline H. Groenland, Monique E. A. M. van Kleef, Jeroen Hendrikse, Wilko Spiering, Jeroen C. W. Siero

**Affiliations:** ^1^Department of Vascular Medicine, University Medical Center Utrecht, Utrecht University, Utrecht, Netherlands; ^2^Department of Radiology, University Medical Center Utrecht, Utrecht University, Utrecht, Netherlands; ^3^Spinoza Centre for Neuroimaging Amsterdam, Amsterdam, Netherlands

**Keywords:** endovascular baroreflex amplification, hypertension, blood oxygenation level-dependent, functional magnetic resonance imaging (fMRI), cerebral blood flow (CBF)

## Abstract

**Background:**

Endovascular baroreflex amplification (EVBA) by implantation of the MobiusHD is hypothesized to lower blood pressure by decreasing sympathetic activity through the mechanism of the baroreflex. In the present exploratory study we investigated the impact of MobiusHD implantation on central sympathetic nerve circuits and cerebral blood flow (CBF) in patients with resistant hypertension.

**Materials and methods:**

In thirteen patients, we performed blood oxygenation level-dependent functional magnetic resonance imaging (BOLD fMRI) at rest and during Valsalva maneuvers, before and 3 months after EVBA. Data were analyzed using a whole-brain approach and a brainstem-specific analysis. CBF was assessed using arterial spin labeling MRI.

**Results:**

Resting-state fMRI analysis did not reveal significant differences in functional connectivity at 3 months after EVBA. For the Valsalva maneuver data, the whole-brain fMRI analysis revealed significantly increased activation in the posterior and anterior cingulate, the insular cortex, the precuneus, the left thalamus and the anterior cerebellum. The brainstem-specific fMRI analysis showed a significant increase in BOLD activity in the right midbrain 3 months after EVBA. Mean gray matter CBF (partial volume corrected) decreased significantly from 48.9 (9.9) ml/100 gr/min at baseline to 43.4 (13.0) ml/100 gr/min (*p* = 0.02) at 3 months.

**Conclusions:**

This first fMRI pilot study in patients with resistant hypertension treated with EVBA showed a significant increase in BOLD activity during the Valsalva maneuver in brain regions related to sympathetic activity. No notable signal intensity changes were observed in brain areas involved in the baroreflex circuit. Future randomized controlled studies are needed to investigate whether the observed changes are directly caused by EVBA.

**Clinical trial registration:**

www.clinicaltrials.gov, identifier: NCT02827032.

## Introduction

Hypertension is one of the major risk factors for cardiovascular disease (CVD), which is a leading cause of morbidity and mortality worldwide (Stanaway et al., 2018). Despite advances in medical therapy, ~10–15% of all hypertensive patients have persistently elevated blood pressure (BP) despite treatment with three or more antihypertensive medications, including a diuretic, and are considered to have resistant hypertension (Judd and Calhoun, 2014). The burden on the health care system, as well as increased cardiovascular mortality related to resistant hypertension, have led to the development of device-based therapies that act beyond standard pharmacological treatment. Since resistant hypertension is characterized by increased sympathetic nerve activity, the sympathetic nervous system with, in particular, the carotid baroreceptor has become one of the most important targets for device-based therapy (Esler, 2014). Endovascular baroreflex amplification (EVBA), a novel device-based hypertension treatment, is assumed to lower BP by decreasing sympathetic activity through the mechanism of the baroreflex (Spiering et al., 2017; van Kleef et al., 2018). With this therapy, a self-expandable nitinol device (MobiusHD) is implanted unilaterally in the carotid sinus, where the mechanosensitive nerve fibers of the baroreceptor are situated (Rees and Jepson, 1970). It is assumed to change the geometric shape of the carotid artery, thereby increasing pulsatile vessel wall strain and baroreceptor firing, which could eventually lead to a reduction in BP (Andresen et al., 1978). The first-in-human study of EVBA showed sustained, clinically meaningful BP reductions with an acceptable safety profile up to 3 years following MobiusHD implantation (van Kleef et al., 2022). However, the central hemodynamic and functional mechanism by which EVBA is thought to lower BP has never been studied. Therefore, the present exploratory study aims to evaluate the effect of MobiusHD implantation on central sympathetic nerve circuits and cerebral blood flow in patients with resistant hypertension. The first aim is to evaluate potential changes in functional connectivity of the salience network, a circuit involved in sympathetic regulation, in resting state. The second aim is to evaluate change in brain (stem) response to large BP fluctuations induced by the Valsalva maneuver, a sympathetic nervous system stimulating task. Lastly, since the MobiusHD implant changes the geometric properties of the carotid sinus which may influence local and/or cerebral blood flow, the final objective is to assess change in cerebral blood flow (CBF) after MobiusHD implantation.

## Materials and methods

### Study design and population

This study was designed as a single-center sub-study of the CALM-DIEM study (Controlling And Lowering blood pressure with the MobiusHD—Defining Efficacy Markers). The CALM-DIEM study [registered at www.clinicaltrials.gov (identifier NCT02827032)] is an open-label, single-arm study designed to investigate the performance of the MobiusHD system in patients (18–80 years of age) with primary resistant hypertension (defined as a 24-h mean ambulatory systolic BP above 130 mmHg on a stable regimen of at least 3 antihypertensive medications, including a diuretic). Main exclusion criteria were: hypertension secondary to an identifiable and treatable cause other than sleep apnea; any plaque, ulceration or stenosis in the carotid artery or the aortic arch; and carotid artery lumen < 5.00 mm or > 11.75 mm or too much tapering at the planned location for implantation. Supplementary Material 1 provides further details on the in- and exclusion criteria of the study. Patients participating in the University Medical Center Utrecht (UMCU) between November 2016 and November 2018 that consented to undergo additional physiological measurements and MRI were included in this sub-study. The effects of MobiusHD implantation on muscle sympathetic nerve activity (MSNA) and baroreflex sensitivity (BRS) are described separately (van Kleef et al., 2021). The study was approved by Medical Ethics Committee United (Nieuwegein/Eindhoven, The Netherlands) and all patients gave their written informed consent. Safety was monitored by an independent data safety monitoring board (DSMB). This sub-study of the CALM-DIEM study was added to www.clinicaltrials.gov after start of participant enrollment. All other ongoing and related trials for this intervention have been registered.

### Study procedures

After confirmation of eligibility, antihypertensive medications were discontinued for a period of 2 weeks (beta-blockers required a preceding 2-week tapering scheme). If deemed appropriate, i.e., when escape medication was required during medication washout in a prior outpatient diagnostic program or when patients developed extreme hypertension, calcium channel blockers were prescribed, with a similar dose at baseline and 3 months. Long-acting calcium channel blockers were chosen because they have no or only a minor effect on sympathetic nerve activity, which is largely limited to acute administration (Del Colle et al., 2007). This strategy was implemented to ensure that all measurements were performed in a stable and similar condition for all patients. MRI and physiological measurements were performed on the same day, in the morning, and after a light meal. Patients had to empty their bladder just before the start of the MRI and physiological recording sessions and were discouraged from engaging in vigorous exercise, smoking and drinking alcohol, coffee, tea or other beverages containing caffeine in the 24-h before and during the measurements. After the measurements, patients were asked to resume their original antihypertensive medications and a bilateral carotid angiography was planned. If one of the internal carotid arteries was suitable for implantation, the MobiusHD was implanted on the anatomically best suited side. The procedure and corresponding antithrombotic and anticoagulant therapy have been described in detail before (Spiering et al., 2017). Three months after MobiusHD implantation, antihypertensive medications were discontinued again (keeping escape medication equal compared to before the implantation) and MRI and physiological recording sessions were repeated. To optimize comparability of the two MRI scans, the second MRI was performed at the same time (between 8 and 10 a.m.) as the first MRI.

### Patient characteristics

**Figure 1:**
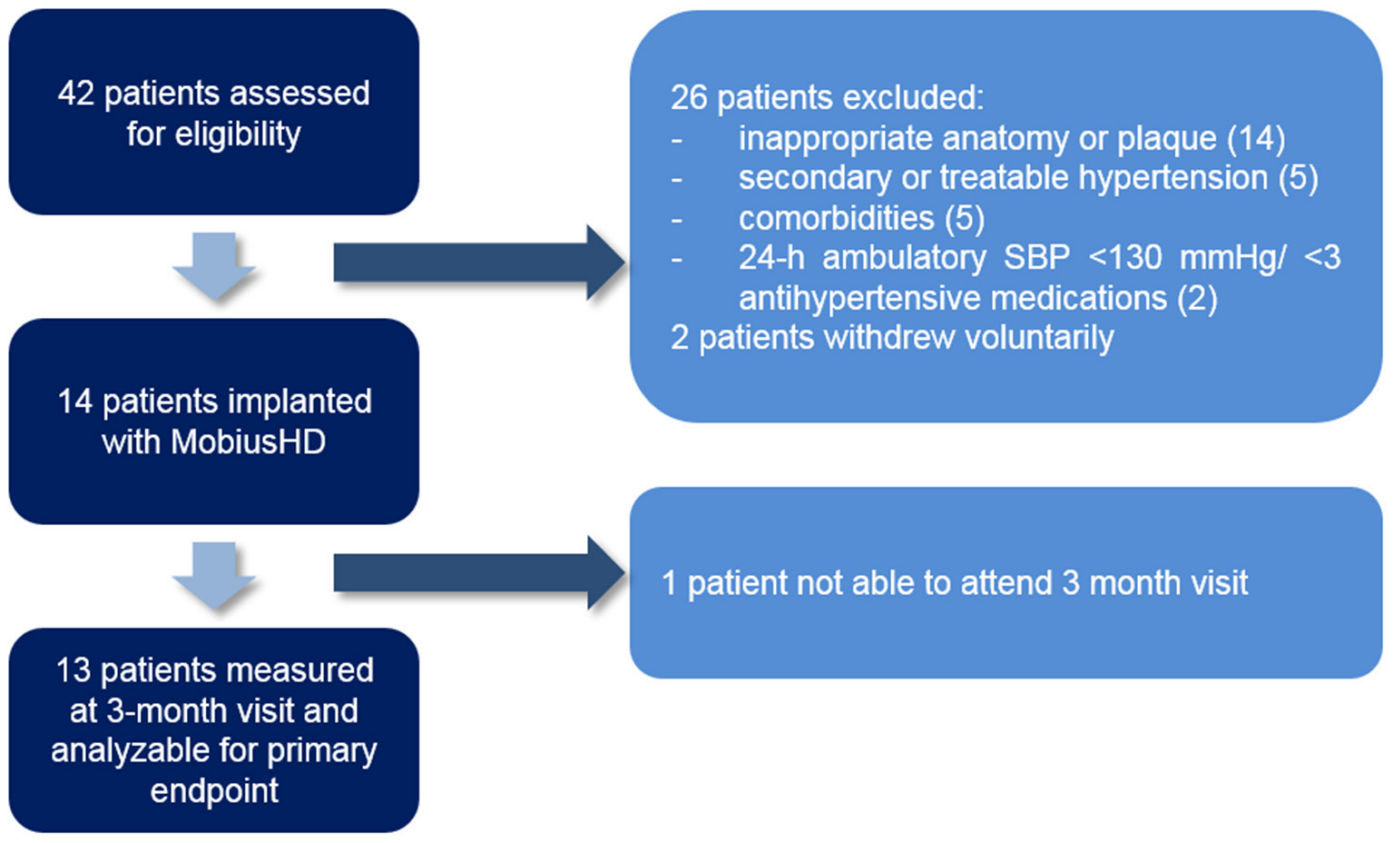
Flow chart of participant progress through the trial. SBP, systolic blood pressure.

Forty-two patients were screened for eligibility (Figure 1), of whom 14 [mean age 52 (±7) years and 10 (77%) patients were men] patients were treated with EVBA. Eight (57%) patients were implanted with the MobiusHD device on the right side and 6 (43%) on the left side. The baseline characteristics of these patients are presented in Table 1. Mean office BP measured in the absence of antihypertensive medication at baseline was 178/107 mmHg (±25/17). Patients used a median of 3 antihypertensive drugs (range 3–5). Prior to MobiusHD implantation, 8 (62%) patients required escape medication. Since one patient was unable to attend the 3-month follow-up visit due to psychosocial issues, which were absent at time of inclusion, 13 patients were included for further analysis.

**Table 1:** Patient characteristics at baseline.

	**Patients implanted**
	***n*** = **14**
Male sex	10 (71%)
Age (years)	52 ± 7
Diabetes mellitus	2 (14%)
Cerebrovascular disease	1 (7%)
Current smoker	2 (14%)
Body mass index (kg/m^2^)	28 ± 5
Estimated GFR (ml/min/1.73m^2^)	81 ± 12
Heart rate (bpm)	79 ± 10
Blood pressure	
Office systolic blood pressure (mmHg)	178 ± 25
Office diastolic blood pressure (mmHg)	107 ± 17
24-h ambulatory systolic blood pressure (mmHg)	156 ± 20
24-h ambulatory diastolic blood pressure (mmHg)	95 ± 15
Medication use	
Number of antihypertensive drugs	3 [3–4]
DDD	5 [3–5]
A+C+D regimen	11 (79%)
A+C+D+MRA regimen	2 (14%)
Escape medication	9 (64%)
Side of mobius HD implantation	
Right side	8 (57%)

*GFR, glomerular filtration rate [calculated with the Chronic Kidney Disease Epidemiology Collaboration (CKDEPI) formula]; DDD, Daily defined doses (calculated using the WHO Collaborating Center for Drug Statistics Methodology DDD Index); A, ACE-inhibitor, angiotensine-II-receptor blocker or direct renin inhibitor; C, calcium channel antagonist; D, diuretic; MRA, mineralocorticoid receptor antagonist. All data in n (%), mean ± standard deviation or median [interquartile range]*.

### fMRI data acquisition

MR imaging was performed in a 3 tesla MR-system (Achieva, Philips Healthcare, Best, The Netherlands) equipped with a 32-channel SENSE head coil (Nova Medical, Wilmington, MA, USA) for reception. Foam pads were placed on the sides of the head to minimize head movement. Heart- and respiratory rates were recorded during the entire scan period using a peripheral pulse unit (PPU) and respiratory belt, respectively. T1-weighted anatomical images were acquired using a three-dimensional (3D) spoiled gradient-echo pulse sequence [whole-brain coverage, matrix size = 256 × 256, 192 sagittal slices, field-of-view (FOV) = 256 × 256 × 192 mm^3^, isotropic voxel size = 1 mm, flip angle = 7°, TR/TE = 8.0/4.5 ms, scan duration 5 min 39 s.]. Pseudo-continuous arterial spin labeling (pCASL) data were acquired using a single-shot 3D gradient- and spin-echo pulse sequence (GRASE); post-label delay time = 1,750 ms, labeling duration = 1,800 ms, labeling plane distance from center FOV = 99.4 mm, whole-brain coverage, matrix size = 80 × 80, 20 axial slices, FOV = 240 × 240 × 140 mm^3^, voxel size = 3 × 3 × 7 mm^3^, flip angle = 90°, TR/TE = 3,000/17.9 ms, SENSE factor = 3, background suppression using two inversion pulses, 30 label-control pairs, scan duration 5 min 24 s. For pCASL CBF quantification, a single proton density-weighted image was obtained for calibration with the same 3D GRASE acquisition parameters but with a TR = 10 s and without labeling and background suppression pulses. Blood oxygenation level-dependent functional MRI (BOLD-fMRI) data were acquired with a T2^*^-weighted multi-echo scan; whole-brain coverage, matrix size = 80 × 80, 41 axial slices, FOV = 240 × 240 × 158 mm^3^, voxel size = 3 × 3 × 3.5 mm^3^, flip angle = 85°, TR = 2,500 ms, TEs = 9.1, 25.2, 41.4 ms, SENSE factor = 3.1, respectively, 116 and 242 volumes were acquired for the resting-state (5 min) and task-based Valsalva maneuvers (10 min 15 s) fMRI scans. During the resting-state scan, patients were instructed not to move and breathe quietly. During the task-based fMRI scan, patients were asked to perform 9 Valsalva maneuvers (each of 15 s) in total in 3 blocks of 3 maneuvers: 2 blocks (i.e., 6 maneuvers) in which a pressure of ~40 mmHg was reached, and 1 block (i.e., 3 maneuvers) in which a pressure of 0–10 mmHg was reached, the “high-pressure Valsalva” (HV) task and “low-pressure Valsalva” (LV) task, respectively (see Supplementary Figure S1). The LV task served as a “control” condition to adjust for changes in neuronal activity due to motor tasks. To execute the Valsalva maneuvers, patients received a mouthpiece that was fixed to ventilation tubing and connected to an analog sphygmomanometer to measure the applied pressure, with a small air leak to prevent a closed glottis. To minimize errors in the performance of the Valsalva maneuver, patients practiced the Valsalva maneuver before the MRI was performed. Moreover, through camera recording of the sphygmomanometer, integrated with an animation video, patients received visual instructions when to start and stop the Valsalva maneuver and whether they applied the correct mouth pressure.

### fMRI data preprocessing

The individual echo images of the multi-echo resting-state and task-based BOLD-fMRI data were first optimally combined using a contrast-to-noise ratio based weighting scheme (Poser et al., 2006). The echo combined data were then corrected for intra-scan motion, linear drift and spatially smoothed using a 5 mm FWHM Gaussian kernel in FSL FEAT (Woolrich et al., 2001; Jenkinson et al., 2012). ICA-AROMA was used to denoise the data and to reduce residual motion-induced artifacts (Pruim et al., 2015). For subsequent whole-brain analysis, denoised fMRI data were spatially registered to the standard MNI (Montreal Neurological Institute) 2 mm^3^ stereotaxic space using FNIRT (Jenkinson et al., 2012). Specifically, the fMRI data was first registered to the high-resolution T1-weighted anatomical image using FLIRT's boundary-based registration and 12 degrees of freedom as an intermediary step.

### fMRI data analysis

#### Resting-state

For the resting-state fMRI analysis, a group independent component analysis (ICA) was performed using MELODIC (Jenkinson et al., 2012). The set of spatial (IC) maps from the group-average analysis was used to generate subject-specific versions of the spatial maps and associated time-series using dual regression (Filippini et al., 2009; Nickerson et al., 2017). In short, first, for each subject, the group-average set of spatial maps is regressed into the subject's 4D space-time dataset. This results in a set of subject-specific time-series, one per group-level spatial IC map. Next, those time-series are regressed into the same 4D dataset, resulting in a set of subject-specific spatial IC maps, one per group-level spatial map. More detailed information on the dual regression method can be found in Nickerson et al. (2017). We then tested for group differences upon MobiusHD implantation treatment using FSL's “Randomize” permutation-testing tool for repeated measures; 5,000 permutations were used. The ICA decomposition was limited to 30 spatially independent components. Motivated by the findings reported by Taylor et al. (2016), we were particularly interested in any treatment-induced changes within the salience network (the network that contained the anterior insular cortex and anterior cingulate cortex).

#### Task-based whole-brain

First level (single subject) analysis was performed on all preprocessed pre- and post-treatment data to estimate the Valsalva-evoked BOLD signal changes, i.e., BOLD activation, by applying a general linear model (GLM) fit using FMRIB Software Library (FSL) FEAT (Jenkinson et al., 2012). The 3 blocks consisting of Valsalva maneuvers were modeled using the recorded mean air pressure values, convolved with a canonical gamma-variate hemodynamic response function incorporating a lag time of 6 s (Woolrich et al., 2001). The GLM model also included 18 slice-wise physiological noise correction parameters, derived from the PPU and respiratory belt data, as confound regressors using RETROICOR (Glover et al., 2000; Harvey et al., 2008). Note that we did not correct for heart rate or respiratory volume as these could have a strong correlation with the Valsalva induced response. The GLM contrast of interest was the HV minus the LV task condition, i.e., a subtraction contrast, which was used to generate z-score statistical activation maps.

Second-level (group) analysis was performed on the first level analysis output and used to compare the BOLD activation prior MobiusHD implantation and 3 months after implantation. Any significant difference in Valsalva-evoked signal changes was determined using fixed-effects linear modeling and a paired Student's *t*-test corrected for multiple comparisons using family-wise error (FWE) correction (using a z-threshold of 3.1 and cluster *p*-value threshold of 0.05) (Woolrich et al., 2004). Significant changes on the group level were overlaid on the MNI atlas image. Cortical areas showing significant changes after treatment were identified using the Talairach anatomical atlas and used as regions-of-interest (ROI) for producing percentage BOLD signal change time courses.

#### Task-based brainstem

A brainstem-specific analysis was performed using a high-resolution template atlas specific for the brainstem and cerebellum from the SUIT toolbox within SPM (Diedrichsen, 2006). After preprocessing, the brainstem and cerebellum were isolated in each subject's T1-weighted anatomical image. Subsequently, the images were registered to MNI space and, together with the BOLD-fMRI data, resliced and smoothed using the SUIT atlas (Diedrichsen, 2006; Hendriks-Balk et al., 2020). Significant changes on the group level were determined using an identical statistical approach as used for the whole-brain analysis. Areas showing significant changes were color-coded and displayed on the reference SUIT atlas.

### Arterial spin labeling CBF analysis

Cerebral blood flow (CBF) was quantified using the procedures as recommended by the consensus paper on arterial spin labeling (ASL) (Alsop et al., 2015). ASL data was preprocessed in terms of denoising and outlier removal using a dual-tree complex wavelet transform (DT-CWT) combined with the non-local means algorithm as reported by Liang et al. (2015) and Dolui et al. (2017), respectively. ASL label and control images were pairwise subtracted to yield cerebral blood flow (CBF) weighted images, subsequent CBF quantification was performed using equation 1 in Alsop et al. (2015) using the acquired proton density-weighted image for calibration. Preprocessing of ASL data was performed using Matlab 2020 (version 2020, Natick USA).

Computed CBF maps were spatially normalized to the standard MNI 2 mm^3^ stereotaxic space using FNIRT (Jenkinson et al., 2012) and using registration to the T1-weighted anatomical image as an intermediate step, similarly as described for the fMRI data. ROI for the cortical gray matter was taken from a tissue segmentation procedure on the T1-weighted anatomical image using FAST (Jenkinson et al., 2012). Mean CBF was calculated for the gray matter ROI, expressed as ml/100 g/min.

### Brain volumetric change assessment

To assess any volumetric changes in gray and white matter and cerebrospinal fluid (CSF) the CAT12 toolbox (www.neuro.uni-jena.de/cat/) of the SPM12 (http://www.fil.ion.ucl.ac.uk/spm/) was used. CAT12 provides an option for longitudinal segmentation using each subject as his/her own template during spatial registration of the T1-weighted anatomical images of the two time points. Subsequently, tissue in each voxel is classified as either gray matter, white matter or CSF, and bias correction is applied to control for intensity non-uniformities. Furthermore, segmentation are modulated by the volume change due to spatial registration. Accordingly, this option is more sensitive to small volumetric changes within the same subject. The default options for CAT12 longitudinal segmentation were used. These include the use of SPM12 tissue probability maps and European brain templates for affine regularization during the initial SPM12 affine registration. The extracted gray and white matter and CSF volumes (in mL) were compared between the two time points; MobiusHD implantation and 3 months after implantation.

### Safety outcomes

Safety outcomes were 30-day major adverse clinical events (death, stroke and/or myocardial infarction), periprocedural device-related serious events (carotid artery rupture, dissection, aneurysm, stenosis or occlusion), serious adverse events and unanticipated adverse device effects.

### Statistics

Statistical analyses were performed with R, version 3.1.1 (R Development Core Team, Vienna, Austria). Data were presented as mean ± standard deviation (SD). Since this is a pilot study, no formal sample size calculation was performed. Statistical analyses on the raw time courses of the BOLD signal changes were performed using a one-way ANOVA. A paired Student's *t*-test was applied to test significance of gray and white matter volume changes and gray matter ASL CBF changes. Pearson's r correlation test was used to test correlation between change in CBF and change in BP. A *p*-value of 0.05 was taken as threshold for significance.

### Role of the funding source

The study sponsor (Vascular Dynamics, Inc.) was involved in study design of the main study, data monitoring and central storage of the data. The study sponsor was not involved in design of the sub-study and had no role in data analysis, data interpretation or manuscript preparation. The authors had full access to the study data and had final responsibility for the decision to submit the paper for publication.

## Results

### Resting-state functional connectivity

**Figure 2:**
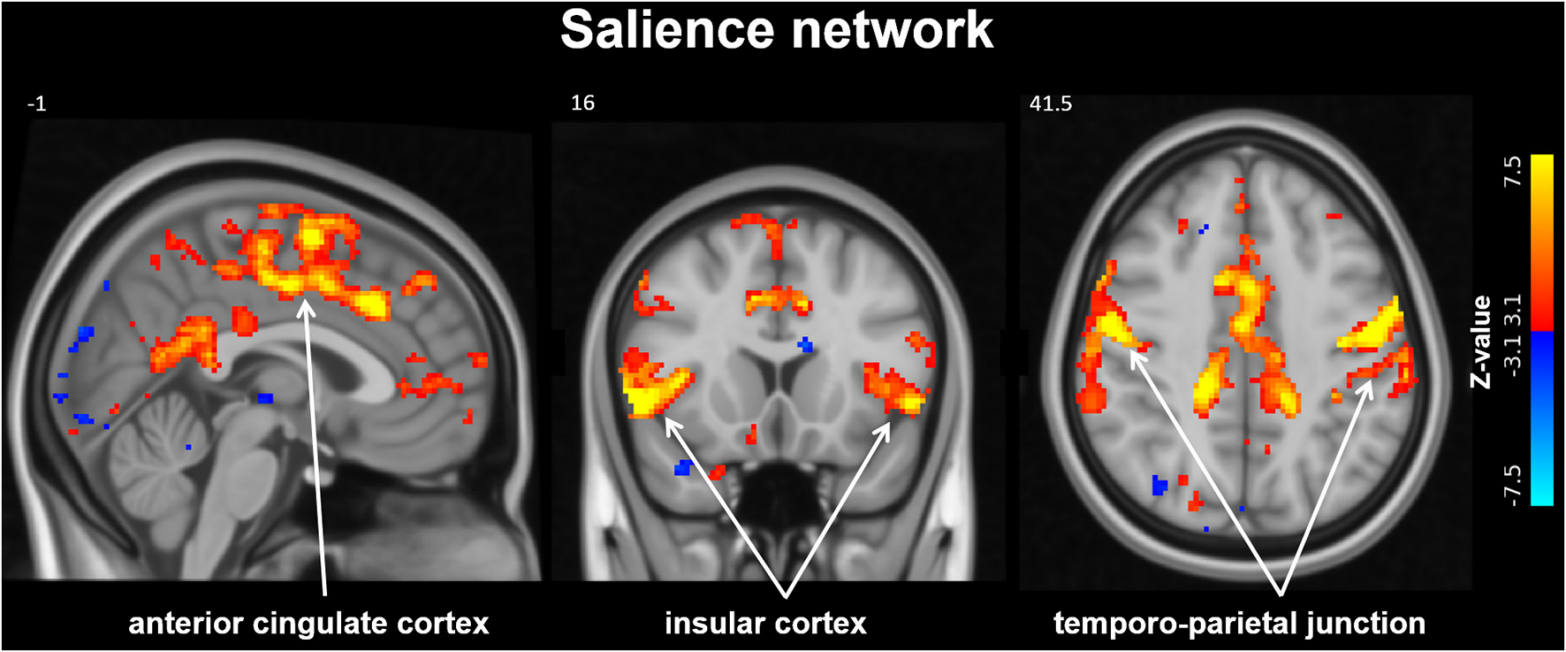
Salience Network identified by resting-state analysis. The salience network (SN) identified by group independent component analysis (ICA). Slice locations in Montreal Neurological Institute space indicated are indicated at the top left of each slice.

From group ICA resting-state fMRI analysis the salience network was identified (see Figure 2), however the dual regression results did not detect significant differences within this network 3 months after MobiusHD implantation.

### Task based fMRI signal intensity changes

#### Whole-brain analysis

**Figure 3:**
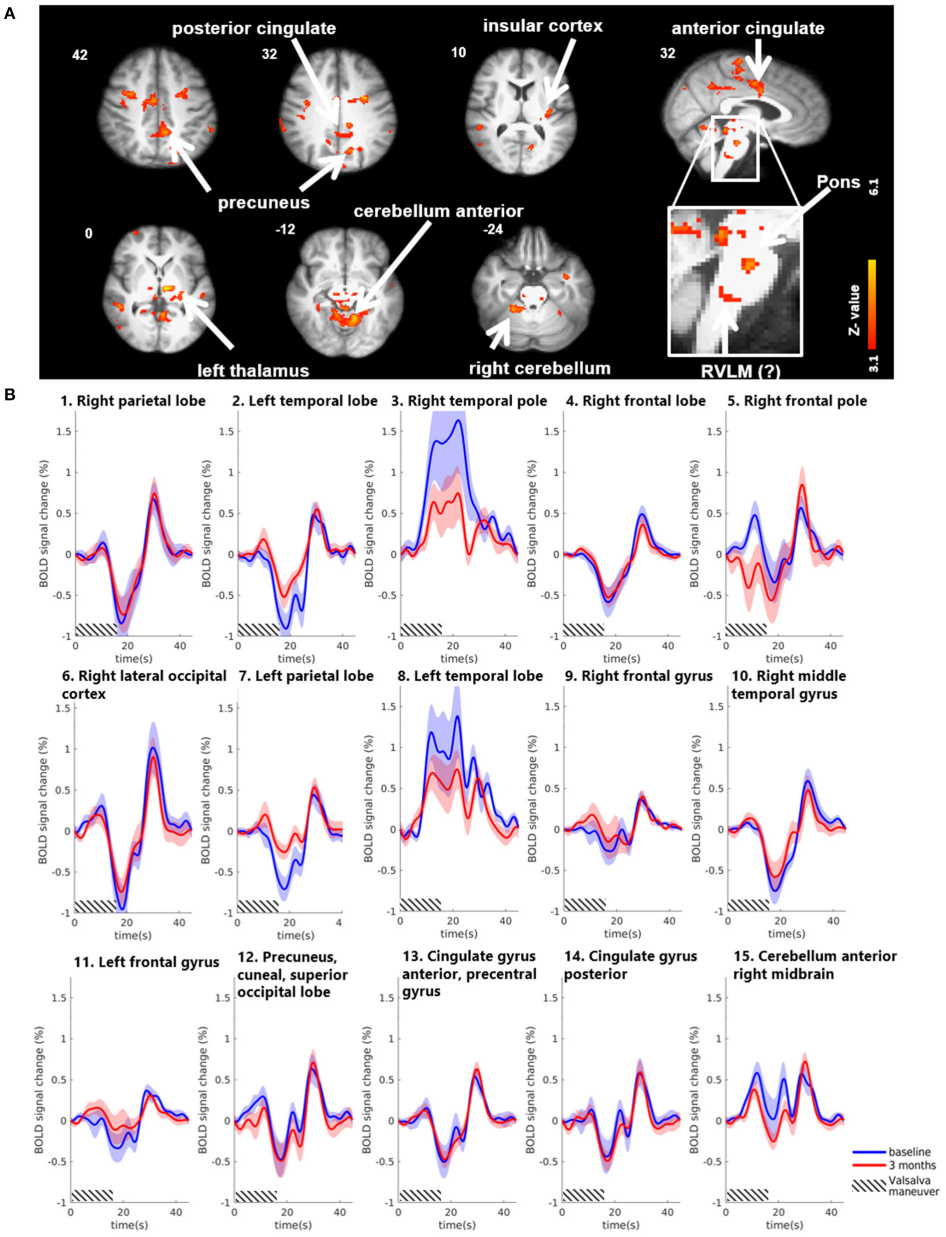
Significant BOLD signal intensity changes in cortical brain regions activated by the Valsalva maneuver. **(A)** Significant activated clusters resulting from the whole-brain analysis with the contrast high Valsalva maneuver pressure (~40 mmHg) minus low Valsalva maneuver pressure (0–10 mmHg) (*n* = 13; threshold of 3.1 for *p* < 0.05) overlaid onto the T1 reference image of SPM12. Data was corrected for motion, physiological noise and scanner drift. Slice locations in Montreal Neurological Institute space indicated are indicated at the top left of each slice. **(B)** Group-averaged BOLD responses (mean ± Standard Error in shaded areas) upon the high-pressure Valsalva maneuver at baseline (blue) and 3 months after implantation (red). Responses are shown for cortical brain regions that showed significant differences for the subtraction contrast HV minus LV task condition 3 months after implantation. The 6 high-pressure Valsalva maneuvers are averaged and normalized at the subject level before averaging at group level (*n* = 13). Except for the temporal lobe regions, the BOLD response shows a biphasic time course upon the high-pressure Valsalva maneuver.

Group results for the contrast HV minus LV revealed significantly increased activations in several brain regions 3 months after MobiusHD implantation compared to baseline (Figure 3). These regions comprised the posterior and anterior cingulate, the insular cortex, the precuneus, the left thalamus and the anterior cerebellum. Moreover, the whole-brain analysis also revealed significant brainstem activations located in the pons (see Table 2 for MNI cluster coordinates, Z-scores and cluster sizes, and Figure 3A for these clusters overlaid onto the T1 reference image of SPM12). Figure 3B shows the BOLD responses in the local maxima of these clusters during the high-pressure Valsalva maneuver.

**Table 2:** Significant changes in BOLD fMRI signal intensity during Valsalva maneuver 3 months after MobiusHD implantation.

**Anatomical structure**	**x**	**y**	**z**	**Z-score**	**Cluster size**	**Cluster no**
Whole-brain analysis						
Right parietal lobe – supramarginal gyrus	64	−40	32	4.16	84	1
Left temporal lobe – superior gyrus	−54	−18	−4	4.13	89	2
Right temporal pole – uncus	30	4	−34	4.54	94	3
Right frontal lobe – post central gyrus	44	−24	36	4.3	95	4
Right frontal pole	20	70	−6	4.65	100	5
Right lateral occipital cortex	52	−68	4	3.95	102	6
Left parietal lobe – inferior lobule	−64	−44	26	5.3	127	7
Left temporal pole – superior temporal gyrus	−34	6	−34	4.71	191	8
Right frontal gyrus	30	2	42	4.83	206	9
Right middle temporal gyrus	50	−40	−2	5.16	241	10
Left frontal gyrus	−32	2	34	5.29	346	11
Precuneus, cuneal, superior occipital lobe	−14	−68	32	4.61	445	12
Cingulate gyrus anterior, precentral gyrus	−2	−24	64	5.17	594	13
Cingulate gyrus posterior, precuneus	−4	−42	44	5.34	674	14
Left cerebellum anterior	−10	−50	−10	6.14	2,014	15
Left thalamus	−6	−16	0	6.1	2,014	15
Brainstem, midbrain, pons, insula, left cerebellum anterior lobe	6	−26	−28	5.21	2,014	15
Brainstem-specific analysis						
Right midbrain	14	−20	−10	4.68	457	1
Right cerebellum anterior lobe – lingual	2	−46	−17	4.67	832	2
Left cerebellum anterior lobe	−12	−50	−21	4.19	1,062	3

*All cerebral and brainstem areas encompassed in the cluster were identified using the Talairach atlas and the Harvard-Oxford Cortical/Subcortical Structural atlas. Active regions after p < 0.05 threshold are reported in MNI standard or SUIT space coordinates with the corresponding z-values for the local maxima; higher z-values indicate higher correlation between task and voxel activation*.

#### Brainstem-specific analysis

**Figure 4:**
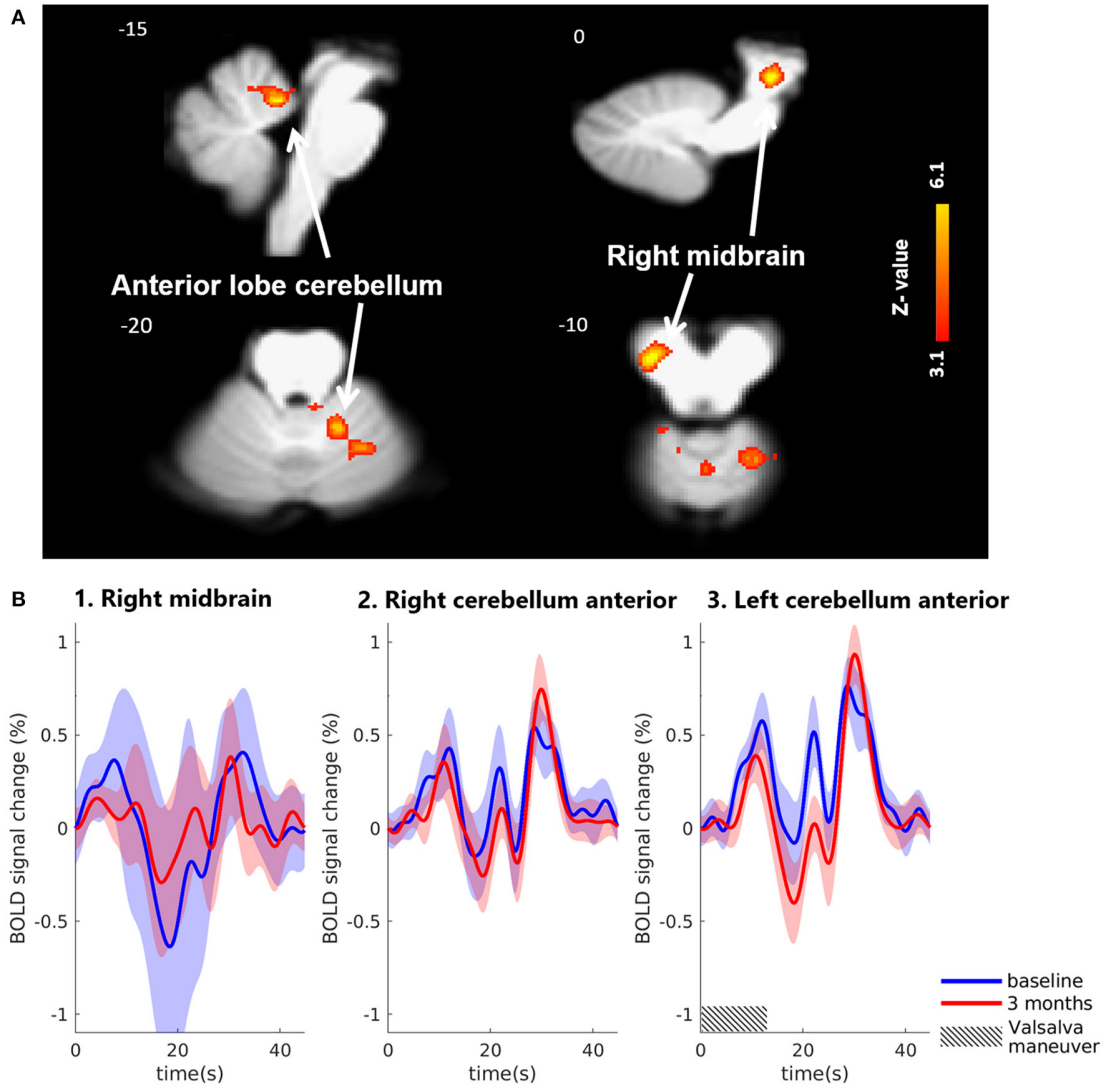
Significant BOLD signal intensity changes in brainstem regions activated by the Valsalva maneuver. **(A)** Significant activated clusters resulting from the brainstem-specific analysis with the contrast high Valsalva maneuver pressure (~40 mmHg) minus low Valsalva maneuver pressure (0–10 mmHg) (*n* = 13; threshold of 3.1 for *p* < 0.05) overlaid onto the T1 reference image of SPM12. Data was corrected for motion, physiological noise and scanner drift. Slice locations in Montreal Neurological Institute space indicated are indicated at the top left of each slice. **(B)** Group-averaged BOLD responses (mean ± Standard Error in shaded areas) upon the high-pressure Valsalva maneuver at baseline (blue) and 3 months after implantation (red). Responses are shown for cortical brain regions that showed significant differences for the subtraction contrast HV minus LV task condition 3 months after implantation. The 6 high-pressure Valsalva maneuvers are averaged and normalized at the subject level before averaging at group level (*n* = 13).

**Figure 5:**
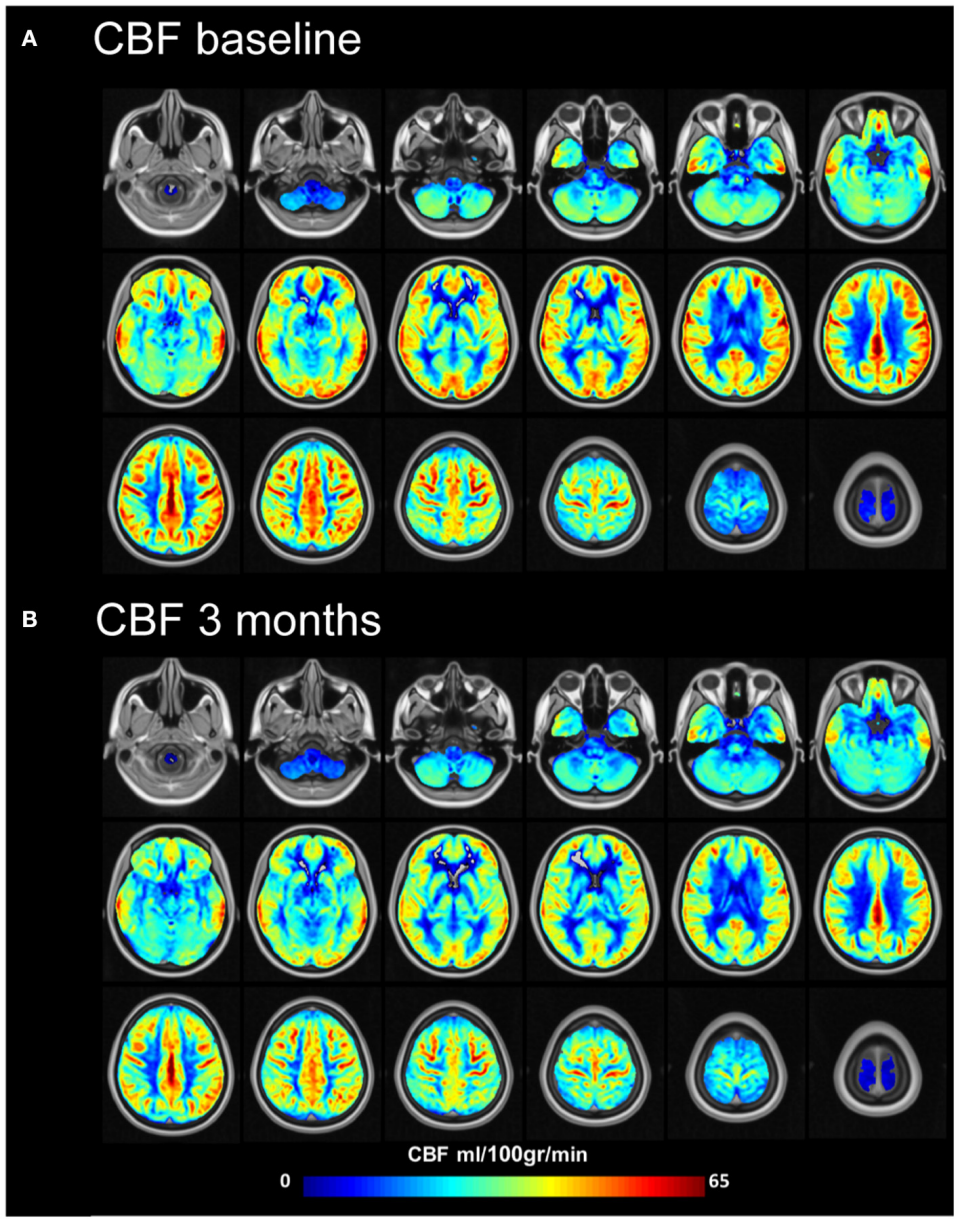
Mean cerebral blood flow (ml/100 gr/min) at baseline and 3 months after MobiusHD implantation. Group cerebral blood flow (CBF) maps before **(A)** and 3 months after **(B)** Endovascular Baroreflex Amplification (EVBA). Data of subjects were averaged for display. Representative brain sections are shown.

Using the brainstem-specific analysis, one cluster of voxels in the brainstem showed an increased BOLD response 3 months after MobiusHD implantation compared to baseline (see Figure 4 and Table 2 for the MNI cluster coordinates, Z-scores and cluster size). This cluster lies within the right side of the diencephalon, and is partly overlapping with the thalamus according to Duvernoy's Atlas of the Human Brain Stem and Cerebellum (Naidich et al., 2009). The significant activation in the pons observed in the whole-brain analysis was not observed in the brainstem-specific analysis. No significant changes in signal intensity occurred in other brainstem regions. Figure 5B shows the BOLD responses in the local maxima of these clusters during the high-pressure Valsalva maneuver.

### Brain volumetric changes

Absolute gray and white matter volumes did not change significantly during the study period {gray matter: 667 mL [standard deviation (SD) 57] at baseline vs. 668 mL (SD 61) at 3 months, *p* = 0.78, white matter: 559 mL (SD 79) at baseline vs. 556 mL (SD 75) at 3 months, *p* = 0.46} (see Table 3).

**Table 3:** Absolute brain volumes at baseline and follow-up.

	**Volumes at**	**Volumes at**	***p*** **-value**
	**baseline (mL)**	**3 months (mL)**	
Gray matter	668 (57)	667 (61)	0.78
White matter	559 (79)	556 (75)	0.46
Total CSF	348 (62)	351 (63)	0.3

*Volumes are given as median and standard deviation (in parentheses). Values are rounded to the nearest integer. Volume changes are calculated from unrounded data. CSF, Cerebrospinal fluid*.

### Cerebral blood flow

Compared to baseline, mean gray matter CBF (partial volume corrected) decreased from 48.9 (9.9) ml/100 gr/min to 43.4 (13.0) ml/100 gr/min (*p* = 0.02) (Supplementary Table S1 and Figure 5) 3 months after MobiusHD implantation. This decrease was not significantly correlated with BP change (Pearson's r correlation coefficient = −0.3, *p* = 0.3) (Supplementary Figure S2). It is possible that the MobiusHD implant influences the ASL labeling efficiency due to the implant position in one of the carotid artery sinuses. To evaluate this, we compared the gray matter CBF between the implanted side vs. the non-implanted side (i.e., left vs. right hemisphere) 3 months after implantation. We found that gray matter CBF in the hemisphere fed by the carotid with the Mobius HD was not significantly different compared to the non-implanted side; mean gray matter CBF (partial volume corrected) 44.1 (13.3) ml/100 gr/min and 45.6 (12.0) ml/100 gr/min (*p* = 0.24), respectively (see Supplementary Tables S2, S3).

### Blood pressure

Mean office BP (measured after medication washout) decreased by −14/−6 mmHg (95% CI −27 to −2/−15 to 3). Mean 24-h ambulatory BP (ABPM) (measured while patients were on medication) did not decrease significantly (−3/−4 mmHg, 95% CI −13 to 7/−12 to 4) (Supplementary Table S4).

### Safety outcomes

During the study, one patient (7%) experienced a major adverse clinical event occurred after MobiusHD implantation on the left side (Supplementary Table S5). This patient developed ischemia in the right putamen (as confirmed by an diffusion-weighted MRI) with accompanying symptoms of vertigo 1 day post-procedure. The DSMB attributed this event to extreme hypotenstion that occurred post-procedure: office systolic BP dropped from 189/112 mmHg at baseline to 100/50 mmHg in the days after the procedure. No periprocedural device-related serious events were observed. Post-procedural groin bleeding occurred in two patients (14%) and required intervention by means of a femostop. In one patient this resulted in prolonged hospitalization and was therefore labeled as a serious adverse event. Other adverse events that were related or possibly related to the device or procedure were: temporary decrease in kidney function (eGFR decrease of >10%, *n* = 3; 21%), pain at the puncture site (*n* = 2; 14%), dizziness (*n* = 1; 7%), epistaxis and axillary hematoma (*n* = 1; 7%), headache (*n* = 1; 7%), and periprocedural chest pain without typical electrocardiographic changes or elevated cardiac biomarkers (*n* = 1; 7%). Unanticipated adverse device effects were not observed during the study.

## Discussion

In this exploratory study, we analyzed the BOLD-fMRI signal changes in resting-state and during the Valsalva maneuver in patients with resistant hypertension treated with EVBA. Three months after MobiusHD implantation, the resting-state analysis did not reveal changes in functional connectivity of the SN. Also, we did not observe changes in BOLD signal activity in the specific areas of the baroreflex pathway in response to the Valsalva maneuver. However, we did observe a significant increase in fMRI signal intensity in other cerebral and brainstem regions. Finally, our most striking observation was a significant decrease in CBF compared to baseline.

To the best of our knowledge, this is the first study investigating the central (sympathetic) nerve circuits before and after MobiusHD implantation by functional MRI in patients with resistant hypertension. Therefore, a direct comparison of our findings with previous literature is not possible.

In the present study, we did not observe changes in functional connectivity of the SN after EVBA. Since the strength of resting-state functional connectivity of the SN has been positively related to muscle sympathetic outflow (Taylor et al., 2016) we hypothesize that the absence of change in resting-state functional connectivity in our study may indicate no change in sympathetic outflow 3 months after MobiusHD implantation. This hypothesis is supported by the lack of a significant decrease in mean 24-h ABPM and muscle sympathetic nerve activity (van Kleef et al., 2021) and the low proportion of treatment responders. Alternatively, the absence of changes in SN connectivity could be due to the limited statistical power of this study. Increasing the sample size or measuring at high magnetic field strength could therefore be future directions.

Previous studies in hypertensive patients treated with electrical devices, also designed to trigger the baroreflex pathway, such as the Rheos system and the Barostim *neo*, did not show significant changes in baroreflex sensitivity (BRS) (Heusser et al., 2010, 2016). Because the mechanism of action of the MobiusHD implant is assumed to be comparable to these devices, we expect the baroreflex sensitivity after MobiusHD implantation to remain intact. The lack of significant changes in BOLD signal activity in brainstem areas associated with the baroreflex pathway in our study does suggest that the cardiovascular compensatory mechanisms in response to the Valsalva maneuver, and thus the baroreflex function, remain unaffected after MobiusHD implantation. The observation that additional measurements of the sympathetic BRS in the same study population also did not show significant BRS changes, supports this hypothesis (van Kleef et al., 2021).

BOLD signal intensity changes during the Valsalva maneuver in other brain areas were most pronounced in the cingulate cortex, anterior insula, the anterior cerebellum, and the right midbrain. These regions have been associated with cardiorespiratory control in humans (King et al., 1999; Banzett et al., 2000; Parsons et al., 2001; Evans et al., 2002; Henderson et al., 2002; Harper et al., 2003; Macey et al., 2003; Macefield et al., 2006). However, whether the observed changes actually indicate a change in the cerebral response to large BP changes, induced by the Valsalva maneuver after MobiusHD implantation, remains unclear. Given the difference in results from the whole-brain and brainstem specific analysis, this could have been a type I error.

The decrease in CBF after MobiusHD implantation found in the present study is concerning. Decreased cerebral perfusion has been associated with a reduction in cortical thickness, acceleration of brain atrophy, and an increased risk of Alzheimer's disease (Alosco et al., 2013). Several mechanisms may explain the observed decrease in CBF. First, as shown in previous studies, chronically high BP is associated with a decline in CBF over time (Beason-Held et al., 2007; Muller et al., 2012). As MobiusHD implantation did not significantly lower the mean 24-h ABPM in our study population, study participants remained exposed to high BP for the duration of the study. Given the lack of existing evidence and the absence of a control group, it remains unknown whether this prolonged exposure to elevated BP fits the natural decline in CBF. Second, MobiusHD implantation may have induced such a large BP drop resulting in reduced CBF that, in the short term, could not sufficiently be compensated by cerebral autoregulation (Zhang et al., 2007). However, since we did not observe a significant correlation between BP change and change in CBF, this explanation seems unlikely. Third, the observed decrease in CBF could also be the result of the MobiusHD implant in one of the carotid artery sinuses, potentially affecting the ALS labeling efficiency and thus the CBF quantification for the associated hemisphere. However, additional analyses to evaluate this hypothesis showed symmetric CBF change. Finally, a potential explanation for the observed decrease in CBF could be poor reliability and reproducibility of ASL measurements in patients with resistant hypertension resulting in false-positive findings. However, previous studies in various populations all showed good reliability and reproducibility (Jahng et al., 2005; Gevers et al., 2009; Xu et al., 2010; Yang et al., 2019; Lin et al., 2020), which makes this explanation less likely.

A major strength of this study is that it is the first to examine the effects of EVBA on the central nervous system in patients with resistant hypertension. Thereby, this study provides important information and sets a foundation for further research into the effects of EVBA. Further strengths of the present study include the elimination of several factors that influence the sympathetic nervous system, such as the implementation of medication washout period, elimination of caffeine and nicotine use, and MR-data acquisition at an equal time of the day at baseline and 3 months. Another strength is the application of brainstem-specific analysis with the SUIT toolbox combined with correction for several physiological noise parameters, which increased the precision of both the acquisition and the analysis of the functional images of the brainstem regions. In addition, we used a low-pressure Valsalva maneuver as a control condition to further filter out noise induced by movement. Finally, the use of arterial spin labeling, which is one of the most reliable methods to quantify cerebral perfusion, is an important strength of the present study (Alsop et al., 2015).

Important methodological limitations to this study need to be addressed. First, we acknowledge the small sample size and absence of a control arm of patients with resistant hypertension that were not treated by EVBA. Consequently, the associations between EVBA and all study endpoints should be interpreted with caution as this is a non-randomized study in which period effects, regression to the mean, Hawthorne effects [a non-specific treatment effect; a change in behavior as a motivational response to the interest, care, or attention received through observation and assessment in the study (Sedgwick and Greenwood, 2015)], placebo effects, and a lack of statistical power most likely affected the results. Second, due to the incompatibility of the MobiusHD device in a 7 tesla MRI scanner, MRI imaging in this study was established using a field strength of 3 tesla. This has resulted in a lower spatial resolution which hinders a spatially detailed analysis of the brainstem region. Third, we did not measure simultaneously fMRI and muscle sympathetic nerve activity or BP, which impeded us to directly relate BOLD signal changes to sympathetic outflow as was done in previous studies (Macefield et al., 2006; Henderson et al., 2012). Fourth, we did not perform 24-h ABPM after antihypertensive medication washout, in order to limit the burden of measurements for the study participants (ABPM was already performed before medication washout). Since office BP measurement is more susceptible to measuring error than ABPM (Xu et al., 2010), the off-medication BP effects observed in the present study are probably less accurate. Fifth, we applied the Valsalva maneuver to stimulate the baroreflex pathway. Although this is a commonly applied approach when investigating the baroreflex, this method requires the active participation of the patient and could have resulted in substantial motion artifacts. In order to minimize these motion artifacts, we trained each subject to perform the maneuver while concentrating on keeping his or her head stationary before scanning and placed foam pads on either side of the head during the MRI scan. In addition, we applied a low-pressure Valsalva control condition and performed motion correction on the image sets of all subjects and further reduced the noise by performing brainstem-specific analysis using the SUIT toolbox. Nevertheless, it should be noted that the reported locations of the significant clusters, particularly within the brainstem, may not fully correspond to the actual brain (stem) regions. Finally, the long-term changes in BOLD signal might differ from the current results and should be investigated in future studies.

## Conclusions

In conclusion, this first exploratory fMRI study did not identify significant changes in functional connectivity in the SN after EVBA which could suggest that there is no change in the central nerve circuits associated with the sympathetic outflow. Moreover, during the Valsalva maneuver, no significant BOLD signal changes were observed in brainstem areas involved in the baroreflex circuit, which could suggest that MobiusHD implantation does not alter the compensatory function of the baroreflex. However, we did observe a small decrease in gray matter CBF 3 months after EVBA, which might be the result of MobiusHD implantation. Future randomized, sham-controlled studies with a larger sample size are needed to investigate whether the observed effects are causally related to MobiusHD implantation.

## Data availability statement

The data that support the findings of this study are available from the corresponding author upon reasonable request.

## Ethics statement

The study involved human participants and was reviewed and approved by the Medical Ethics Committee United (Nieuwegein/Eindhoven, Netherlands). The participants provided their written informed consent to participate in this study.

## Author contributions

EG and MK: conceptualization, data curation, formal analysis, investigation, methodology, visualization, and writing—original draft. JS: conceptualization, data curation, formal analysis, investigation, methodology, software, supervision, visualization, and writing—review and editing. JH: conceptualization and writing—review and editing. WS: conceptualization, funding acquisition, supervision, and writing—review and editing. All authors gave final approval and agree to be accountable for all aspects of work ensuring integrity and accuracy.

## Funding

This study received funding from Vascular Dynamics, Inc. The funder was not involved in the study design, collection, analysis, interpretation of data, the writing of this article, or the decision to submit it for publication.

## Conflict of interest

EG and MK were indirectly paid from a research grant by Vascular Dynamics, Inc. WS is a consultant for Vascular Dynamics and has received a research grant from Vascular Dynamics. The remaining authors declare that the research was conducted in the absence of any commercial or financial relationships that could be construed as a potential conflict of interest.

## Publisher's note

All claims expressed in this article are solely those of the authors and do not necessarily represent those of their affiliated organizations, or those of the publisher, the editors and the reviewers. Any product that may be evaluated in this article, or claim that may be made by its manufacturer, is not guaranteed or endorsed by the publisher.

## Abbreviations

ASL: arterial spin labeling
ABPM: ambulatory blood pressure measurement
BOLD: blood oxygenation level-dependent
BP: blood pressure
CVLM: caudal ventrolateral medulla
EVBA: endovascular baroreflex amplification
fMRI: functional magnetic resonance imaging
MRI: magnetic resonance imaging
NTS: nucleus tractus solitarius
RVLM: rostral ventrolateral medulla.
