# Supplementary material for: The effect of endovascular baroreflex amplification on central sympathetic nerve circuits and cerebral blood flow in patients with resistant hypertension: A functional MRI study
Source: Front Neuroimaging. 2022 Jul 25;1:924724. doi: 10.3389/fnimg.2022.924724 (PMC10406262; doi:10.3389/fnimg.2022.924724)
Supplement: Supplementary file 1 [file Data_Sheet_1.docx]

**Supporting information**

# The effect of endovascular baroreflex amplification (EVBA) on central sympathetic nerve circuits and cerebral blood flow in patients with resistant hypertension: a functional MRI study

Eline H. Groenland^1^*, MD; Monique E.A.M. van Kleef^1^*, MD, PhD; Jeroen Hendrikse^2^; MD, PhD; Wilko Spiering^1^, MD, PhD, Jeroen C.W. Siero^2,3^, PhD; ^1^Department of Vascular Medicine, University Medical Center Utrecht, Utrecht University, the Netherlands; ^2^Department of Radiology, University Medical Center Utrecht, Utrecht University, the Netherlands; ^3^Spinoza Centre for Neuroimaging Amsterdam, Amsterdam, The Netherlands

*These authors contributed equally to this work

**Corresponding author**

Jeroen Siero, PhD, Dr. ir; Department of Radiology; Center for Image Sciences, University Medical Center Utrecht; P.O. Box 85500; 3508 GA Utrecht; The Netherlands; Phone: +31 (0)88 7553262; Email: [J.C.W.Siero@umcutrecht.nl](mailto:J.C.W.Siero@umcutrecht.nl)

**Table of contents**

Supplemental Files

[S1 File - Detailed description of study in- and exclusion criteria 3](#_Toc75807122)

[Supplemental Tables 6](#_Toc75807121)

[S1 Table - Individual gray matter CBF at baseline, follow-up and changes after 3 months 6](#_Toc75807122)

[S2 Table - Cerebral blood flow in left hemisphere 7](#_Toc75807123)

[S3 Table – Cerebral blood flow in right hemisphere 8](#_Toc75807124)

[S4 Table – Blood pressure and heart rate before and after MobiusHD implantation 9](#_Toc75807125)

[S5 Table – Overview of safety outcomes that occurred within 3 months after implantation](#_Toc75807126) 10

[Supplemental Figures 1](#_Toc75807127)1

[S1 Fig. – Example scheme of the Valsalva maneuvers performed during task-based fMRI.](#_Toc75807128) 11

[S2 Fig. - Correlation between cerebral blood flow and 24h blood pressure 1](#_Toc75807130)2

# Supplemental Files

## Supplemental File 1 – Detailed description of study in- and exclusion criteria

**Inclusion criteria**

Candidates for this study must meet the following criteria to be enrolled:

***Screening***

1. ≥ 18 years of age and ≤ 80 years of age;
2. Diagnosed with primary resistant hypertension;
3. Mean 24-hour systolic ambulatory BP ≥130mmHg following at least 30 days on a stable antihypertensive medication regimen (no changes in medication or dose), and no more than 28 days prior to implantation.

**Exclusion criteria**

Candidates will be ineligible for enrolment in the study if any of the following conditions are identified:

***Screening***

1. An inability provide written informed consent;
2. Known or clinically suspected baroreflex failure or autonomic neuropathy;
3. Known significant aortoiliac or common femoral artery disease that will prohibit safe femoral access;
4. Hypertension secondary to an identifiable and treatable cause other than sleep apnea (e.g. hyperaldosteronism, renal artery stenosis, pheochromocytoma, Cushing's disease, coarctation of the aorta, hyperparathyroidism and intracranial tumor);
5. Treatable cause of resistant hypertension including, but not limited to improper BP measurement, volume overload and pseudotolerance (excessive sodium intake, volume retention from kidney disease, inadequate diuretic therapy), drug-induced or other causes (non-adherence, inadequate doses, inappropriate combinations, NSAIDs, COX-2 inhibitors, cocaine, amphetamines, other drugs, sympathomimetics, oral contraceptives, adrenocortical steroids, cyclosporine, tacrolimus, erythropoietin, excessive liquorice (including some chewing tobacco), ephedra, ma huang, bitter orange, and excessive alcohol intake);
6. Arm circumference greater than 46 cm and/or BMI ≥45;
7. Chronic atrial fibrillation or recurrent atrial fibrillation with episode within the last 12 months;
8. History of bleeding complications with dual antiplatelet therapy in the past or known uncorrectable bleeding diathesis;
9. Current use of anticoagulation therapy, other than dual antiplatelet medications; examples include vitamin K antagonists and direct-acting oral anticoagulants including apixaban, rivaroxaban, dabigatran and edoxaban;
10. Peptic ulcer disease with documented active ulcer or bleeding within the last year;
11. History of allergy to contrast media that cannot be managed medically;
12. Persistent symptomatic orthostatic hypotension (>20/10 mmHg);
13. Persistent symptomatic syncope documented to be related to hypertension within the last 6 months;
14. History of myocardial infarction or unstable angina within the past 3 months;
15. History of cerebral vascular accident (stroke or TIA) within the past year, and NIHSS >5 or mRS >1;
16. Chronic kidney disease (eGFR calculated by the Modification of Diet in Renal Disease equation <45 ml/min);
17. Prior carotid surgery, therapeutic radiation, or endovascular stent placement in either carotid region;
18. Severe valvular or structural heart disease (excluding left ventricular hypertrophy);
19. Severe chronic obstructive pulmonary disease (requiring 24-hour oxygen or oral steroids), asthma, or severe pulmonary hypertension;
20. Uncontrolled diabetes mellitus with HbA1c ≥10%;
21. Active infection within the last month requiring antibiotics;
22. Uncontrolled co-morbid medical condition, including mental health issues, that would adversely affect participation in the trial;
23. Co-morbid condition that reduces life expectancy to less than 1 year;
24. Planned surgery or other procedure within the next 6 months requiring cessation of antiplatelet medications;
25. Pregnant or lactating females. For females of child-bearing potential, a positive pregnancy test within 7 days of the pre-randomization screening or refusal to use a medically accepted method of birth control for the duration of the trial;
26. Carotid duplex studies demonstrating obstructive carotid disease, plaque, ulceration or >150 micron intima-media thickness (IMT) at the site of implantation and/or proximal to the carotid artery bulb and ≥50% disease distal to the carotid artery bulb, including the intracranial circulation;
27. Significant obstructive vascular disease, calcification or plaque of aortic arch and great vessels by ultrasound, computed tomography angiography (CTA) or magnetic resonance angiography (MRA);
28. Renal artery stenosis >50% or systolic gradient >10 mmHg in borderline cases diagnosed by renal artery imaging in the last 36 months. Acceptable renal artery imaging modalities include renal duplex, CTA, MRA, and selective or nonselective renal angiography depending on trial site diagnostic standards;
29. Internal carotid artery (ICA) lumen diameters <5 mm or >12.5 mm within the planned location of the implant placement via CTA or MRA. Evidence of landing zone restrictions, such as inadequate length, vessel tapering, and/or vessel curvature that would preclude safe placement of the implant;
30. Enrolled in a concurrent clinical trial of an investigational drug or device that has not yet reached its primary endpoint;
31. Unable or unwilling to fulfil the protocol follow-up requirements;
32. Subject is a prisoner or member of other vulnerable population.
33. Use of anti-hypertensive medications directly acting on the sympathetic nervous system, that cannot be discontinued safely;
34. Uncontrolled or involuntary movements disturbing microneurography, such as tremors, fasciculations and chorea;
35. Absence or paralysis of both legs;
36. Polyneuropathy or clinical suspicion for autonomic nervous system dysfunction;
37. Known claustrophobia;
38. Metallic implants, prostheses or other foreign bodies causing potential artefacts obscuring the visibility of signals from the site of MobiusHD implantation during MRI;
39. Cochlear implants, pacemakers, neurostimulators, stents or grafts at risk of malfunction due to the magnetic field;
40. Underlying conditions that prohibit a Valsalva maneuver: i.e. aortic stenosis, cardiac arrhythmia, glaucoma, and/or retinopathy.

***Day of Procedure - Angiographic***

1. Evidence of any carotid plaque, ulceration or any stenosis on selective carotid angiography performed in orthogonal views. Luminal diameters will be assessed to exclude subjects with ICA lumen diameters <5 mm or >11.75 mm within the planned location of the device placement;
2. Any angiographic evidence of plaque or ulceration in the aortic arch and/or the supra aortic vasculature;
3. Inappropriate anatomy of the carotid bifurcation for deployment of the MobiusHD, including, but not limited to, tortuosity of the extracranial vessels and significant angulation of the common carotid artery bifurcation;
4. Type III arch or horizontal take-off of the left carotid from the innominate and any significant calcification of the carotid bulb.

# Supplemental Tables

## S1 Table - Individual gray matter CBF (ml/100gr/min) at baseline, follow-up and changes after 3 months

|  | | | | | | |
| --- | --- | --- | --- | --- | --- | --- |
|  | **Not partial volume corrected** | | | **Partial volume corrected** | | |
| **Patient no.** | **Baseline** | **Follow-up** | **∆ CBF** | **Baseline** | **Follow-up** | **∆ CBF** |
| 1 | 38.9 | 31.3 | -7.7 | 55.0 | 48.0 | -7.0 |
| 2 | 39.0 | 24.3 | -14.7 | 50.8 | 35.9 | -14.9 |
| 3 | 50.0 | 49.5 | -0.6 | 59.5 | 58.8 | -0.7 |
| 4 | 45.9 | 48.4 | 2.5 | 58.8 | 58.4 | -0.4 |
| 5 | 31.8 | 29.8 | -2.0 | 46.8 | 43.6 | -3.1 |
| 6 | 41.2 | 44.1 | 2.8 | 56.8 | 57.6 | 0.8 |
| 7 | 42.2 | 39.6 | -2.6 | 56.2 | 54.5 | -1.7 |
| 8 | 16.0 | 11.7 | -4.3 | 23.9 | 18.2 | -5.7 |
| 9 | 34.1 | 47.2 | 13.1 | 49.8 | 57.3 | 7.5 |
| 10 | 31.6 | 27.6 | -4.0 | 47.0 | 40.7 | -6.3 |
| 11 | 27.3 | 22.6 | -4.7 | 42.0 | 33.5 | -8.5 |
| 12 | 45.3 | 23.4 | -21.9 | 58.4 | 34.4 | -24.0 |
| 13 | 38.6 | 29.4 | -9.2 | 52.0 | 43.1 | -8.9 |
| Mean±SD | 37.1±9.4 | 33.0±12.3 | -4.1±8.5 | 50.5±1.0 | 44.9±12.9 | -5.6±7.8* |

CBF = cerebral blood flow, SD = standard deviation, *p<0.05

## ****S2 Table – Cerebral blood flow in left hemisphere****

|  |  | **Gray matter** | | | | **White matter** | | | |
| --- | --- | --- | --- | --- | --- | --- | --- | --- | --- |
|  |  | **Non-PVC** |  | **PVC** |  | **Non-PVC** |  | **PVC** |  |
| Patient no. | Side MobiusHD | Baseline | Follow-up | Baseline | Follow-up | Baseline | Follow-up | Baseline | Follow-up |
| 1 | right | 38.9 | 31.7 | 53.8 | 48.0 | 28.3 | 23.8 | 22.8 | 19.2 |
| 2 | right | 38.0 | 23.5 | 50.0 | 34.8 | 27.3 | 15.9 | 21.8 | 12.2 |
| 3 | left | 51.5 | 50.3 | 60.1 | 59.1 | 34.7 | 33.0 | 27.7 | 24.9 |
| 4 | right | 45.5 | 48.5 | 58.0 | 57.7 | 32.2 | 35.0 | 25.8 | 28.0 |
| 5 | left | 32.1 | 30.7 | 46.5 | 44.4 | 25.3 | 23.7 | 19.8 | 18.7 |
| 6 | left | 42.8 | 46.1 | 56.5 | 56.9 | 30.7 | 34.0 | 24.2 | 27.9 |
| 7 | left | 42.6 | 39.8 | 56.3 | 54.7 | 27.9 | 29.3 | 20.4 | 23.4 |
| 8 | right | 16.7 | 11.8 | 25.1 | 18.7 | 11.9 | 7.8 | 9.2 | 5.3 |
| 9 | right | 35.3 | 48.7 | 50.9 | 58.8 | 25.2 | 35.8 | 20.0 | 29.3 |
| 10 | right | 31.7 | 27.7 | 47.0 | 40.9 | 23.4 | 21.7 | 18.5 | 18.0 |
| 11 | left | 27.3 | 17.4 | 42.2 | 25.4 | 19.9 | 14.3 | 15.1 | 11.5 |
| 12 | right | 45.8 | 24.8 | 57.9 | 36.4 | 30.0 | 15.9 | 24.6 | 12.5 |
| 13 | left | 38.3 | 29.4 | 51.7 | 43.1 | 24.8 | 18.0 | 18.9 | 13.2 |
| Mean±SD | | 37.4±9.1 | 33.1±12.6 | 50.5±9.3 | 44.5±13.1 | 26.3±5.8 | 23.7±9.1 | 20.7±4.8 | 18.8±7.6 |

PVC = partial volume corrected, SD = standard deviation

## ****S3 Table – Cerebral blood flow in right hemisphere****

|  |  | **Gray matter** | | | | **White matter** | | | |  |
| --- | --- | --- | --- | --- | --- | --- | --- | --- | --- | --- |
|  |  | **Non-PVC** |  | **PVC** |  | **Non-PVC** |  | **PVC** |  |  |
| Patient no. | Side MobiusHD | Baseline | Follow-up | Baseline | Follow-up | Baseline | Follow-up | Baseline | Follow-up |  |
| 1 | right | 38.7 | 30.8 | 55.8 | 47.9 | 27.4 | 20.6 | 21.5 | 15.3 |  |
| 2 | right | 39.6 | 24.8 | 51.4 | 36.7 | 26.9 | 16.0 | 20.2 | 11.2 |  |
| 3 | left | 48.4 | 48.5 | 58.6 | 58.4 | 32.5 | 29.9 | 25.2 | 22.6 |  |
| 4 | right | 46.2 | 48.1 | 59.4 | 58.8 | 32.5 | 33.7 | 25.5 | 26.8 |  |
| 5 | left | 31.7 | 29.1 | 47.2 | 43.1 | 24.7 | 22.4 | 19.0 | 17.3 |  |
| 6 | left | 39.8 | 42.2 | 57.1 | 58.2 | 27.5 | 30.7 | 21.8 | 25.3 |  |
| 7 | left | 41.9 | 39.3 | 56.1 | 54.3 | 26.9 | 27.6 | 19.8 | 22.0 |  |
| 8 | right | 15.2 | 11.4 | 22.6 | 17.6 | 9.5 | 7.3 | 6.6 | 4.7 |  |
| 9 | right | 32.9 | 45.6 | 48.6 | 55.8 | 21.4 | 31.5 | 15.3 | 23.9 |  |
| 10 | right | 31.5 | 27.5 | 47.0 | 40.5 | 21.9 | 20.7 | 16.9 | 16.8 |  |
| 11 | left | 27.3 | 27.2 | 41.9 | 40.6 | 19.8 | 21.2 | 15.0 | 16.3 |  |
| 12 | right | 44.6 | 22.0 | 58.7 | 32.6 | 28.5 | 14.1 | 23.1 | 11.1 |  |
| 13 | left | 38.5 | 29.3 | 52.1 | 43.2 | 25.2 | 17.4 | 18.6 | 12.5 |  |
| Mean ±SD | | 36.6±8.9 | 36.6±11.2 | 32.8±10.0 | 50.5±12.2 | 45.2±6.0 | 25.0±7.8 | 22.6±5.0 | 19.1±6.5 | |

PVC = partial volume corrected, SD = standard deviation

## S4 Table - Blood pressure and heart rate before and after MobiusHD implantation

|  | **baseline** | **3 months** | **p-value** |
| --- | --- | --- | --- |
| Office^*^ SBP (mmHg) | 182 (6) | 168 (6) | 0.027 |
| Office^*^ DBP (mmHg) | 112 (5) | 105 (5) | 0.150 |
| 24-h Ambulatory^ǂ^ SBP (mmHg) | 155 (6) | 152 (7) | 0.512 |
| 24-h Ambulatory^ǂ^ DBP (mmHg) | 96 (4) | 93 (5) | 0.353 |
| HR (bpm) | 74 (3) | 69 (4) | 0.074 |

*Office blood pressure was measured after medication washout. ^ǂ^ 24-h Ambulatory blood pressure was measured while patients were on medication. numbers represent means (SE), p-values are obtained from paired t-tests HR = heart rate, BP = blood pressure, SBP = systolic BP, DBP = diastolic BP

## ****S5 Table – overview of safety outcomes that occurred within 3 months after implantation****

|  | **Number of patients** | **% of implanted patients** |
| --- | --- | --- |
|  |  |  |
| 30-day major adverse clinical events |  |  |
| *Death* | 0 | 0% |
| *Stroke* | 1 | 7% |
| *Myocardial infarction* | 0 | 0% |
| Periprocedural device-related serious events | 0 | 0% |
| Unanticipated adverse device effects | 0 | 0% |
| Serious adverse events |  |  |
| *Groin bleeding requiring prolonged hospitalization* | 1 | 7% |
| Non-serious adverse events* |  |  |
| *Groin bleeding* | 1 | 7% |
| *Temporary decrease in kidney function*˟ | 3 | 21% |
| *Pain at puncture site* | 2 | 14% |
| *Dizziness* | 1 | 7% |
| *Epistaxis and axilary hematoma* | 1 | 7% |
| *Headache* | 1 | 7% |
| *Periprocedural chest pain* | 1 | 7% |
|  |  |  |
| *only those related or possibly related to the device or procedure or with relatedness unknown | | |
| ˟decrease in eGFR of >10% |  |  |

# Supplemental Figures

## S1 Fig. – Example scheme of the Valsalva maneuvers performed during task-based fMRI

**
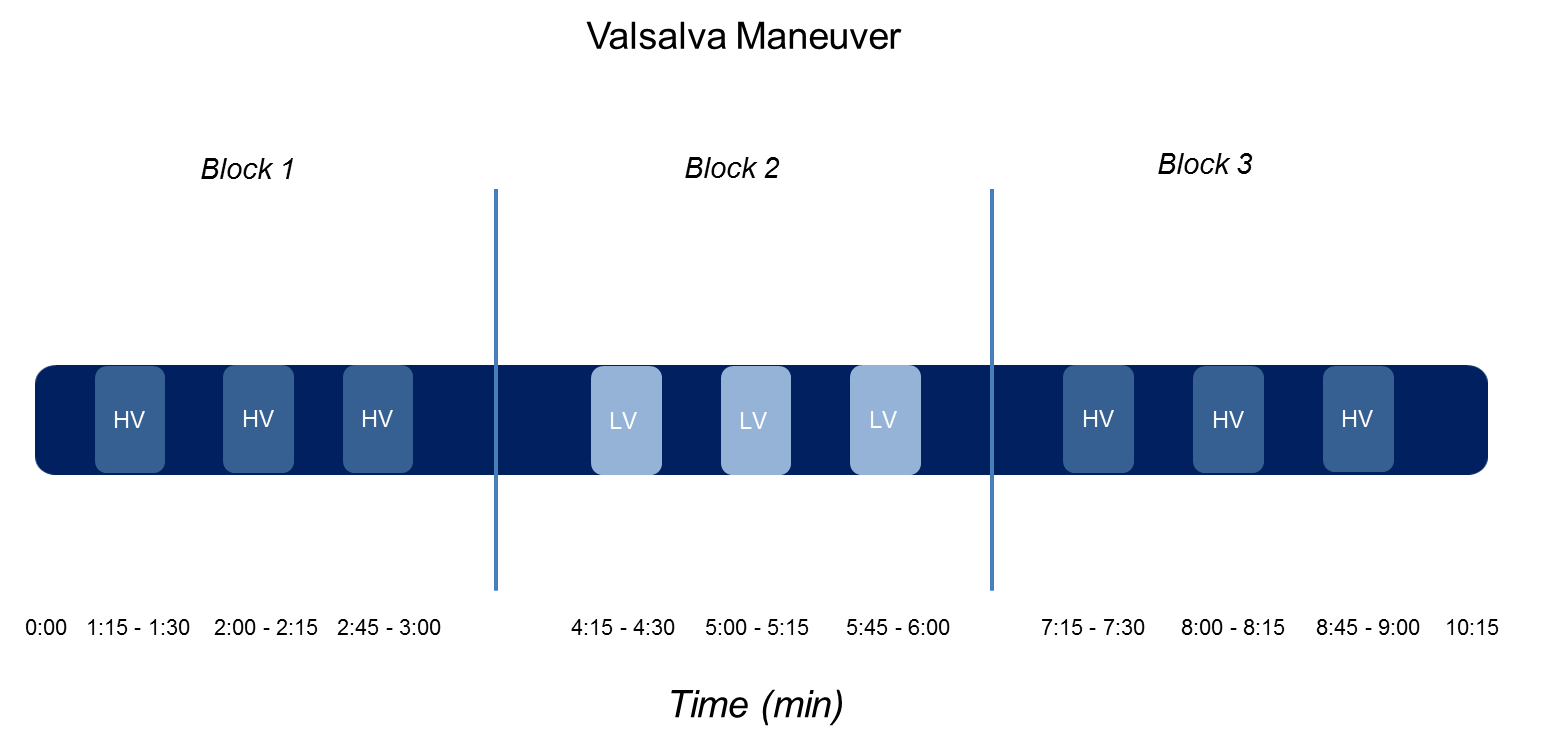
**

During the task-based fMRI scan, patients were asked to perform 9 Valsalva maneuvers (each of 15 s) in total in 3 blocks of 3 maneuvers: 2 blocks (i.e. 6 maneuvers) in which a pressure of ~40 mmHg was reached, and 1 block (i.e. 3 maneuvers) in which a pressure of 0-10 mmHg was reached as a control condition, the “high-pressure Valsalva” (HV) task and “low-pressure Valsalva” (LV) task, respectively.

## S2 Fig. - Correlation between cerebral blood flow and 24h blood pressure

**
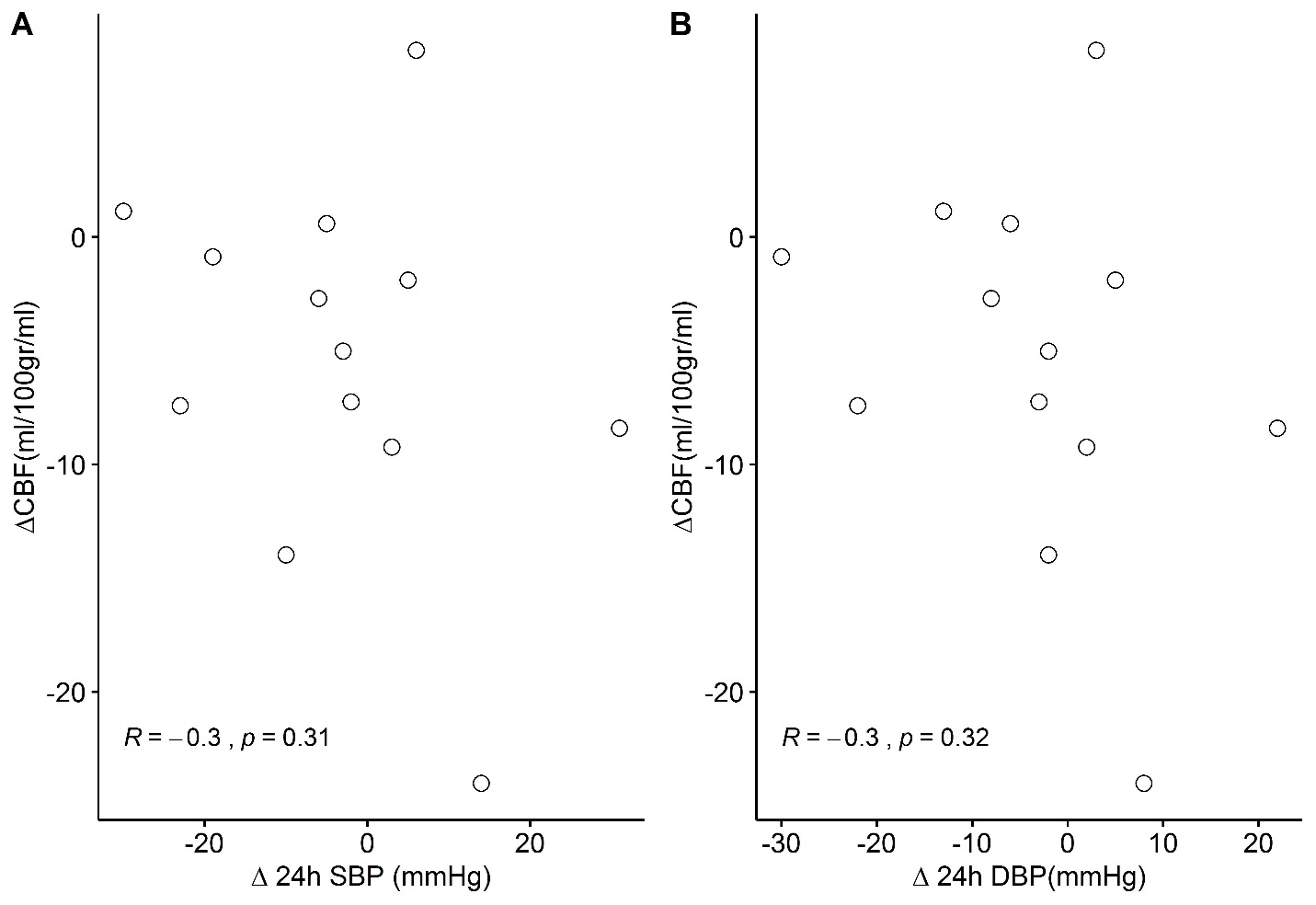
**

Correlation between change in cerebral blood flow and change in 24h systolic (A) and diastolic (B) blood pressure 3 months after EVBA. To assess the association between change in blood pressure and change in cerebral blood flow, the Pearson’s r correlation coefficient was used. R = correlation coefficient, SBP= systolic blood pressure, DBP = diastolic blood pressure, CBF = Cerebral Blood Flow
